# Advances in multiplex PCR: balancing primer efficiencies and improving detection success

**DOI:** 10.1111/j.2041-210X.2012.00215.x

**Published:** 2012-10

**Authors:** Daniela Sint, Lorna Raso, Michael Traugott

**Affiliations:** Institute of Ecology, University of InnsbruckTechnikerstraße 25, 6020 Innsbruck, Austria

**Keywords:** diagnostic PCR, PCR optimisation, primer sensitivity, standardise DNA templates, trophic interactions

## Abstract

**1.** Multiplex PCR is a valuable tool in many biological studies but it is a multifaceted procedure that has to be planned and optimised thoroughly to achieve robust and meaningful results. In particular, primer concentrations have to be adjusted to assure an even amplification of all targeted DNA fragments. Until now, total DNA extracts were used for balancing primer efficiencies; however, the applicability for comparisons between taxa or different multiple-copy genes was limited owing to the unknown number of template molecules present per total DNA.

**2.** Based on a multiplex system developed to track trophic interactions in high Alpine arthropods, we demonstrate a fast and easy way of generating standardised DNA templates. These were then used to balance the amplification success for the different targets and to subsequently determine the sensitivity of each primer pair in the multiplex PCR.

**3.** In the current multiplex assay, this approach led to an even amplification success for all seven targeted DNA fragments. Using this balanced multiplex PCR, methodological bias owing to variation in primer efficiency will be avoided when analysing field-derived samples.

**4.** The approach outlined here allows comparing multiplex PCR sensitivity, independent of the investigated species, genome size or the targeted genes. The application of standardised DNA templates not only makes it possible to optimise primer efficiency within a given multiplex PCR, but it also offers to adjust and/or to compare the sensitivity between different assays. Along with other factors that influence the success of multiplex reactions, and which we discuss here in relation to the presented detection system, the adoption of this approach will allow for direct comparison of multiplex PCR data between systems and studies, enhancing the utility of this assay type.

## Introduction

**Fig. 1:**
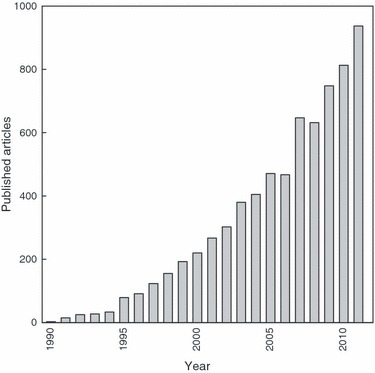
Number of published articles per year found in the Web of Knowledge (Thomson Reuters, New York, NY, USA) when searching for the term ‘multiplex PCR’ (in quotation marks) as topic.

Multiplex PCR systems are increasingly used in biological and medical studies as they allow simultaneous amplification of several DNA fragments within one reaction. This ability to reduce the number of reactions needed to test a sample for different targets helps saving time and money and makes multiplex systems useful especially when large sample numbers have to be screened. Therefore, multiplex PCR is regularly used for examining population genetics and parentage assignment (e.g. Guichoux *et al.* 2011), to investigate trophic interactions (e.g. Harper *et al.* 2005; Macfadyen *et al.* 2009), for molecular species identification (e.g. Staudacher *et al.* 2011a) and community assessment (e.g. Hosseini *et al.* 2007; Albuquerque *et al.* 2009; Gioia *et al.* 2010), as well as in forensic (e.g. Hill, Butler & Vallone 2009) and food safety studies (e.g. Randhawa, Chhabra & Singh 2009). The considerable potential of this method is also reflected in the rapidly rising numbers of publications that have adopted this approach (Fig. 1). Surprisingly, although this method is widely applied, few papers address methodological issues and how to improve and standardise multiplex PCR assays.

Today, various manufacturers offer multiplex PCR kits, often advertising them as ‘ready-to-use’, or ‘no optimisation needed’. While this is usually true for reagents such as buffers (including concentrations of KCl and MgCl_2_), dNTPs or DNA polymerases, it is still necessary to optimise primer concentration or thermocycling conditions to achieve balanced and stable reactions. The primers, especially, play a crucial role as their performance is strongly influenced by characteristics such as internal stability, melting temperature, secondary structure or interference with each other (Apte & Daniel 2003). For example, it is known that fragments targeted by better performing primer pairs will be amplified preferentially (Markoulatos, Siafakas & Moncany 2002) or that amplification efficiency of general primers, which target a range of species, can vary between species (Polz & Cavanaugh 1998; Sipos *et al.* 2007). This leads to unbalanced amplification strength and differing detection limits among targets within and between multiplex PCRs and should be levelled out before application of this type of assay.

The extent of assay optimisation, however, should be adjusted to the intended application to achieve a system optimised to the study’s needs while avoiding unnecessary work, time and costs for overperfectioning a multiplex PCR. One example where balancing primer efficiencies is not so essential is species identification from well-preserved DNA (e.g. Dusfour *et al.* 2007; Lee *et al.* 2008). For this type of application, the amount of template DNA is not a limiting factor and equal signal strength for different targets is not mandatory because only one species-specific fragment [or two, if an internal general control is used alongside (e.g. Mendonca *et al.* 2009; Roques *et al.* 2011)] is expected to be amplified from each tested individual. However, when samples contain only limited amounts of DNA (King *et al.* 2008; Masseret *et al.* 2010), primer sensitivity needs to be balanced to avoid biasing detection rates towards the most sensitive primer pairs (Markoulatos, Siafakas & Moncany 2002). This is also true when the DNA is already degraded, as for example when working with museum material or faeces (Kanuch *et al.* 2007), or when partly digested food remains should be identified (King *et al.* 2008). Additionally, varying primer efficiency can increase allelic dropout in population genetic studies.

So far, the adjustment and sensitivity testing of multiplex PCR is usually performed using a known amount of extracted total DNA of the target organism as template (Hill, Butler & Vallone 2009; Robinson *et al.* 2010; Webster *et al.* 2010; Eitzinger & Traugott 2011; King *et al.* 2011). This is not a problem when single-copy genes are targeted and the signal strength for different loci of a single individual has to be balanced within a reaction (e.g. in microsatellite analysis). However, the approach is not so well suited for targeting multiple-copy genes and/or different species in a multiplex system, as considerable variation in copy numbers of multiple-copy genes can occur within and between species (Kumar & Rai 1990; Herrera *et al.* 2009). This means, although the same amount of total DNA is present for each target, the actual number of template molecules is unknown. It becomes even more problematic when different multiple-copy genes (e.g. mitochondrial and ribosomal genes) are targeted within one reaction. This variation in the amount of target DNA hampers balancing multiplex PCR efficiencies for different targets and usually impedes comparisons between multiplex assays and among studies.

To overcome the problem of varying copy numbers in total DNA extracts, we propose to adjust multiplex PCR sensitivity using standardised DNA templates. This allows balancing multiplex PCR systems for different primer efficiencies as well as to compare and standardise detection sensitivity between assays. Based on a multiplex PCR system that was set up to investigate feeding interactions in a high Alpine arthropod community, we demonstrate, step-by-step, how to generate standardised DNA templates and how to use them to standardise assay sensitivity for different targets. Furthermore, we discuss how this approach is related to other important steps during the set-up of a new multiplex PCR assay.

## Materials and methods

### Case study

We developed a multiplex system to investigate feeding interactions in the foreland of the glacier ‘Rotmoosferner’ (Tirol, Austria); its arthropod community is well studied (Kaufmann 2001). The species community of recently deglaciated areas is relatively simple and dominated by predatory arthropods (Kaufmann 2001; Hodkinson, Webb & Coulson 2002), making glacier forelands ideal study sites to investigate the build-up of food webs. Beside different predators (ground beetles, wolf spiders and harvestmen), collembolans and a rich spectrum of flying insects can be observed in vicinity to the newly ice-free areas of the ‘Rotmoosferner’.

Surface-active invertebrates were caught in dry pitfall traps. Predatory species were individualised, starved for a minimum of 7 days to allow digestion of their gut content, then freeze-killed and subsequently stored in ethanol until DNA extraction. Non-predatory species such as collembolans, which were used for testing the specificity of the new multiplex PCR assay, were directly transferred into ethanol, as were flying insects caught within malaise traps and yellow and grey bowls.

### Multiplex primer design

Identification of the target species was performed by the authors, supported by expert taxonomists for high alpine arthropods from the Institute of Ecology, University of Innsbruck, Austria. Part of the mitochondrial cytochrome c oxidase subunit one gene (COI) was sequenced for all target species using primers LCO1490/HCO2198 (Folmer *et al.* 1994) (GenBank accession numbers JQ746510–JQ746527). The sequences were aligned using BioEdit (Hall 1999), and specific primer pairs were designed using Primer Premier 5 (Premier Biosoft International, Paolo Alto, CA, USA) for the following taxa: *Nebria germari* Heer, *Nebria jockischii* Sturm, *Nebria rufescens* (Stroem), *Oreonebria castanea* (Bonelli) (all Coleoptera: Carabidae); *Pardosa* spp. [targeting *Pardosa nigra* (C.L. Koch), *Pardosa saturatior* Simon and *Pardosa giebeli* (Pavesi)] (Araneae: Lycosidae) and *Mitopus glacialis* (Heer) (Opiliones: Phalangiidae). For Collembola, a group-specific primer combination targeting the 18s rRNA gene is available (Kuusk & Agustí 2008), but it was not working well within our system. Therefore, based on sequences retrieved from GenBank (Table S1), an alternative reverse primer was designed and combined with Col3F (Kuusk & Agustí 2008). During design, primer pairs were balanced for melting temperatures and cross-dimers were avoided where possible. As QIAxcel, an automated capillary electrophoresis system (Qiagen, Hilden, Germany), was used for separation and visualisation of PCR products, amplicon length differences of as little as 20 bp were suitable to differentiate between targets within the multiplex system. QIAxcel produces also electropherograms, where relative fluorescent units (RFU) provide a measure for signal strength of the detected fragments.

### Generating standardised DNA templates

A fragment of COI was amplified from each predator using the general primers mentioned previously (Folmer *et al.* 1994). *Pardosa nigra* DNA was used as template for the general *Pardosa* primer in the multiplex system, as this is the most abundant species in the investigated system. For Collembola, primers 18sL0466/18sR1100 (Luan *et al.* 2003) were used to amplify part of the 18s rRNA gene. By doing so, a DNA fragment was generated for each target included in the multiplex PCR, which encompassed the specific primer-binding sites. PCR products were cleaned with QIAquick PCR purification kit (Qiagen) following the instructions of the manufacturer, and DNA quantity was measured using Quant-iT™ PicoGreen® (Invitrogen, Paisley, UK). DNA quantities were determined as means from three individual measurements of each product on a VICTOR™*X4* Multilabel Plate Reader (PerkinElmer, Waltham, MA, USA).

The molecular weight of each double-stranded fragment (DS) was calculated from the respective DNA sequence using Eqn 1, where AT is the number of A–T pairs and GC the number of G–C pairs in the fragment.


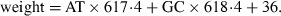
eqn 1

Then, the number of DS fragments per μl was calculated based on the DNA quantity (*q*; in ng μL^−1^) derived from the PicoGreen® measurements for each target using Eqn 2. A computer program combining Eqns 1 and 2 with a tool to extract the numbers of AT and GC pairs from a sequence is available as supporting material (Data S1) or can be downloaded from our web page (http://www.uibk.ac.at/ecology/forschung/biodiversitaet.html.en).


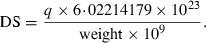
eqn 2

Based on the number of fragments calculated, the PCR products were diluted stepwise to standardise the numbers of DS copies for all targets (10 000–5 copies μL^−1^). These standardised templates (single products and mixtures of the seven targets containing equal numbers of DNA molecules) were used to adjust between the primer pairs for equal amplification within the system and to determine the PCR sensitivity.

### Adjusting the multiplex system to obtain equal amplification efficiency

Before multiplexing, all primer pairs were tested in singleplex PCRs at the estimated optimal annealing temperature to check for correct amplification of the desired fragments. Then, a first provisional multiplex system was tested. Each 10 μL PCR mix contained 1·5 μL DNA template, 1× QIAGEN Multiplex PCR master mix, 0·25× Q-Solution (Qiagen), 5 μg bovine serum albumin (BSA), all primers at a final concentration of 0·2 μM and RNase-Free water (Qiagen) to adjust the volume. Cycling conditions were 15 min at 95 °C, 35 cycles of 30 s at 94 °C, 90 s at 62·5 °C, 60 s at 72 °C and final elongation 10 min at 72 °C. At this step it was also checked, that no additional fragments were produced by any of the primers within the multiplex system. The multiplex system was then tested in a gradient PCR with single extracts and a mix of all targeted taxa to determine the optimal annealing temperature. Finally, primer concentrations were adjusted stepwise by decreasing those pairs that resulted in relatively strong signals and increasing the ones producing too weak bands in steps of 0·1 μM. This led to the final multiplex system, resulting in equal signal strength for all targets when a mix of standardised template DNA (i.e. same number of template molecules per target) was used.

### Testing PCR sensitivity

To estimate the sensitivity of the primer pairs in the multiplex system, the minimum number of DS template copies necessary to amplify a product that resulted in ≥0·1 RFU was identified for all targets under various conditions: (1) only one type of target DNA as template, (2) a mix containing all targets at equal concentrations as template, (3) one type of target DNA as template plus ∼300 ng of non-target DNA (Lithobiidae), (4) an equal mix of all targets plus ∼300 ng of non-target DNA (Lithobiidae). Through the addition of a large amount of non-target DNA, the presence of non-amplifiable consumer DNA was simulated and the influence on assay sensitivity determined.

### Primer specificity

To test whether the primers amplify only with the targeted taxa and that they do not cross-react with DNA from other animals, a wide variety of ground-dwelling and arial arthropods living in or close to recently deglaciated areas was collected. Various trap types (pitfall traps, malaise traps, yellow and grey bowls) together with hand catching were used to collect these taxa. Malaise traps and bowls were filled with saturated salt water with a drop of detergent to break surface tension and emptied on a daily base. A total of 121 DNA extracts from 37 different non-target taxa (mainly family level) plus Collembola of those animals where then used for non-target testing to ensure specificity of the assay within the high Alpine community. The mentioned trap types for collecting non-target animals can be problematic, as cross-contamination between animals might occur (King *et al.* 2008; Greenstone *et al.* 2011) but no other trap type/collection method would have provided us with a sufficient amount of the needed material.

## Results

### Primer efficiency and multiplex PCR

**Fig. 2:**
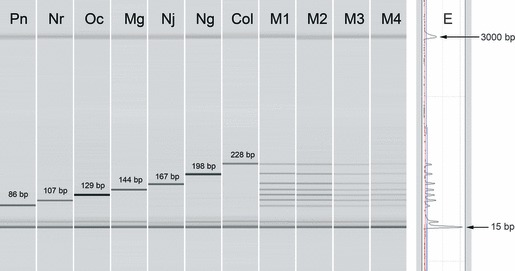
Multiplex PCR conducted with standardised numbers of DNA templates and separated with QIAxcel (Qiagen) where an internal marker (15 and 3000 bp) is run with each sample. Description of Lanes: Pn, *Pardosa nigra*; Nr, *Nebria rufescens*; Oc, *Oreonebria castanea*; Mg, *Mitopus glacialis*; Nj, *Nebria jockischii*; Ng, *Nebria germari*; Col, Collembola; each with 10 000 double-stranded copies as template (tc); M1–M4 standardised DNA mixes. M1, 2100 tc per target; M2, 1000 tc per target; M3, 200 tc per target; M4, 100 tc per target; E, electropherogram of Lane M3. Note: when a single target was present at high concentrations, signal strength was not balanced (e.g. Oc and Ng resulted in stronger signals); however, this did not occur at lower concentrations.

In the first provisional multiplex PCR, all primer pairs were present at equal concentrations (0·2 μM). Under these conditions, primers targeting *N. rufescens*, *N. jockischii* and *M. glacialis* showed the highest DNA amplification efficiency, while those targeting *N. germari* and collembolans were the least efficient. Thus, they were adjusted to the concentrations given in Table 1. The final multiplex system (10 μL PCR mix) contained 1·5 μL DNA template, 1× QIAGEN Multiplex PCR master mix, 0·25× Q-Solution (Qiagen), 5 μg BSA, each primer at its specific concentration (Table 1) and RNase-Free water (Qiagen) to adjust the volume. Cycling conditions were 15 min at 95 °C, 35 cycles of 30 s at 94 °C, 3 min at 60 °C, 1 min at 72 °C and final elongation 10 min at 72 °C. This multiplex system resulted in an even amplification of all seven fragments when the templates of all targets were mixed equally, independent of the overall number of templates (Fig. 2) and unaffected by the presence of non-target DNA.

**Table 1:** Primer pairs designed from COI mtDNA (*Pardosa*, *Mitopus*, *Oreonebria* and *Nebria* species) and 18s rRNA gene (Collembola)

	Targets	Primer names and sequences (5′–3′)	Conc. (μM)	Size (bp)
Multiplex system	*Pardosa* spp.	Pard-sp-S238: CTGTTTATCCTCCTTTAGCATCTAC	0·2	86
		Pard-sp-A239: AGCCCCAGCTAAATGAAGAG		
	*Nebria rufescens*	Neb-ruf-S249: TCAGTCGGAATTACTGCATTAC	0·1	107
		Neb-ruf-A250: GGGTCAAAGAAAGTTGTATTTAAG		
	*Oreonebria castanea*	Ore-cas-S240: CTCTGTTGACTTAGCTATTTTCAGA	0·2	129
		Ore-cas-A241: AATAAAGGTATTCGATCAAAGGA		
	*Mitopus glacialis*	Mit-gla-S243: TATACCCCCCTCTATCAAGAAAT	0·1	144
		Mit-gla-A244: TACCTTGTGTTCGTATGTTGATG		
	*Nebria jockischii*	Neb-joc-S242: GTGAACAGTTTACCCTCCACTG	0·1	167
		Neb-joc-A243: TTCGGTCAAAAGTTATACCAATT		
	*Nebria germari*	Neb-ger-S241: CGAATGAATAATATAAGATTTTGACTT	0·4	198
		Neb-ger-A242: AGCCCCTAAAATTGAAGAAATA		
	Collembola	Col3F: GGACGATYTTRTTRGTTCGT	0·4	228
		Col-gen-A246: TTTCACCTCTAACGTCGCAG		

	*N. germari*	Neb-ger-S256: ATTAGGAAACCCTGGGTCC	1	210
		Neb-ger-A255: AGTTAATGAAGGGGGAAGAAG		

Columns show the primer targets, primer names (S and A denote forward and reverse primers, respectively), primer sequences, the final concentration in the multiplex reaction and the product size. Primer Col3F from Kuusk & Agustí (2008); all other primers designed in the present study. Primer pair S256/A255 was not included in the multiplex, but used to verify *N. germari* amplicons in a singleplex PCR.

### PCR sensitivity

The system proved to be highly sensitive: as little as 20–30 DS templates were sufficient to amplify a detectable (i.e. ≥0·1 RFU) amount of DNA for each target. When lower quantities of templates (down to seven templates per reaction) were tested, still all fragments were detectable, but signal strength was <0·1 RFU for some fragments and replicability was not assured. This was independent of whether the template DNA was added as a single target or in a mix containing all targets. The addition of non-target DNA did not reduce assay sensitivity – still with an estimated number of 20–30 templates per target, a stable reaction was observed. Reducing template numbers further led to increased variability in amplification success.

### Primer specificity

During specificity testing, we detected some non-target organisms that produced fragments at the expected length of our targeted taxa when tested with the multiplex system. By sequencing these DNA products obtained from non-targets with the corresponding primers and comparing them with our target sequences, it turned out that most of these non-target samples were contaminated with target DNA (e.g. collembolan DNA present in extracts of Diptera or Lepidoptera). This contamination most likely occurred within traps, but a few individuals produced unspecific products close to the expected products for *N. jockischii* (167 bp) or *N. germari* (198 bp) (e.g. some Collembola produced a double-band at ∼165 bp with the specific primers). These problems could be solved by retesting positive samples with either the primers for *N*. *jockischii* (S242/A243) or an additional primer pair for *N*. *germari* (S256/A255) (Table 1) in a singleplex reaction. Conditions were the same as for the multiplex PCR, only the primer mix was replaced by 1 μM of either primer pair S242/A243 or S256/A255. Table S2 shows the details of all tested individuals from the specificity test; products that turned out to be contamination are not displayed.

## Discussion

In the present article, we describe a new and easy method to obtain standardised DNA templates that can be used for sensitivity testing and balancing primer efficiencies in multiplex PCR systems. This is a significant improvement compared to the use of genomic DNA extracts as it allows levelling sensitivity within and between assays, independent of the targeted taxa or variation in gene copy numbers. Applying this standardisation technique to the newly developed multiplex assay for Alpine, arthropods allowed us to compensate for different amplification efficiencies between primer pairs, avoiding biased results towards preferentially amplified targets.

Aside from balancing primer efficiency by standardising template DNA concentration, we also want to address other critical steps during the development and optimisation of multiplex PCR systems in the subsequent discussion, following the logical sequence of assay establishment.

### Primer selection and amplicon size

Compared to singleplex PCR, where only one primer pair is present within one reaction, several primer pairs are acting in multiplex PCRs, introducing some extra factors that have to be considered. When planning a multiplex system, primers should be checked during primer design in all combinations to avoid potential formation of primer cross-dimers. Moreover, melting temperatures need to be balanced for all primers to achieve an even performance.

The generated amplicons need to show appropriate size differences that allow one to unambiguously differentiate the fragments in electrophoresis, although the absolute size difference between two neighbouring fragments strongly depends on the method used for separation. On agarose gels, differences have to be rather large (i.e. >30 bp for fragments <300 bp and increasing with fragment size), whereas high resolution capillary electrophoresis systems such as QIAxcel or a DNA sequencer allow separating fragments that are very similar in size (1–20 bp). While QIAxcel detects DNA via an ethidium bromide-stained matrix, DNA sequencers are able to detect several fluorescent markers attached to the amplified DNA in parallel, allowing even differentiation of fragments of the same size as long they are labelled differently. However, this advantage comes at the cost of higher prices for both labelled primers and fragment analysis using a DNA sequencer compared to QIAxcel or standard gel electrophoresis. Modifying primers also can change their properties depending on the type of modification (Guichoux *et al.* 2011; A. Juen, R.A. King & W.O.C. Symondson, personal communication). This means that they have to be optimised in their final form and interchanging of labelling dyes cannot be performed easily once a system is established.

In general, assuring noticeable size differences in amplicons is not a problem when a sufficient amount of fresh tissue is available for DNA extraction (e.g. for species identification), but it becomes challenging when degraded DNA has to be targeted. While in the first case, it is possible to include also longer amplicons in the multiplex system, giving a wider potential range for appropriate primer sites, the latter should preferably include only fragments with less than 300 bp in length (King *et al.* 2008), significantly delimitating flexibility in primer design.

Aside from the separation of the amplicons, the number of primer pairs that can be included in a multiplex system is also limited by the total number of primers used within an assay as each additional primer increases the risk of cross-reactivity between primers and amplification of unwanted DNA fragments.

### Balancing primer efficiency

Once primers are chosen that work together in one reaction without showing cross-reactivity or production of artefacts, it is still very likely that they will differ in efficiency. Therefore, primer concentrations in the reaction mix have to be adjusted for each primer pair to compensate for varying amplification efficiency. Here, capillary electrophoresis systems and DNA sequencers have another advantage: they allow comparing the outcome of different PCRs via electropherograms where the signal strength, given in RFU, is related to the amount of amplified DNA. Thus, an evaluation of whether a multiplex reaction is balanced for all targets is much easier than compared to standard gel electrophoresis.

So far, known amounts of total DNA extracts of target organisms have been used for adjusting primer concentrations and for sensitivity testing (Traugott *et al.* 2008, 2012; Amagliani *et al.* 2009). We think that this is a good strategy in microsatellite analysis, where the templates for all amplicons are different DNA regions within one individual. These target regions are mostly single-copy genes placed on chromosomes that are typically present in fixed numbers per cell, thus all templates for individual primer pairs are present in equal or comparable amounts within each sample. Accordingly, if the reaction is balanced for a few individuals, it will most likely also be so for any further samples. For species identification from fresh tissue, where the amount of template is not limited, the use of total DNA extracts is also well suited for testing a newly developed system. On the other hand, this approach is problematic when DNA of different targets can be co-present within the PCR, for example when species assemblages or unknown numbers of different taxa are targeted. Here, interspecific comparisons or adjustments are impossible as the amount of actual template available for the reaction varies with genome size, and, if targeted, differences in copy numbers of multiple-copy genes can be considerable. This means, although the same amount of total DNA is present for each target, the available number of template molecules will be different (Kumar & Rai 1990; Herrera *et al.* 2009). Examples for such ‘mixed systems’ could be micro-organisms that are targeted for community characterisation (Filteau *et al.* 2011) or diagnostic purposes (e.g. Elnifro *et al.* 2000; Amagliani *et al.* 2009; Hamiduzzaman, Guzman-Novoa & Goodwin 2010; Robinson *et al.* 2010). Other applications where the adjustment of multiplex PCR conditions is necessary are studies investigating trophic interactions (e.g. King *et al.* 2010; Pitzer *et al.* 2011; Staudacher *et al.* 2011b; Traugott *et al.* 2012). Molecular analysis of feeding interactions summarises all aforementioned problems and uncertainties: the targeted DNA is an unknown mixture of small quantities of degraded DNA and usually consumer DNA is excessively present as well. In this case, multiple-copy genes are mostly targeted to enhance the detection probability (King *et al.* 2008). Given that different prey-taxa are often detected within one multiplex assay, interspecific differences in copy numbers of multiple-copy genes are likely to be the standard situation and an important issue when adjusting multiplex PCR conditions as mentioned previously.

To overcome these hurdles, we propose the new approach outlined here, where standardised amounts of the DNA templates are used. By equalling the number of template molecules available for amplification, different primer efficiencies in the multiplex reaction can be determined and subsequently balanced by changing the concentration of each primer pair individually. This allows bringing amplification success for each target to a comparable level. Consequently, it helps to minimise assay-induced bias in detection rates, diminishing the risk of drawing wrong conclusions in diagnostic and ecological studies. If general or group-specific primers are involved in molecular studies, this approach is not so easy to apply, as mismatches between primers and certain target species might occur if the primer is not located in a conserved region. In this case, the sensitivity of a reaction can vary between species targeted by the same primer pair dependent on the number and the location of the mismatches. Here, we suggest to determine primer efficiency separately for several targets to measure the range in sensitivity. The adjustment of the primers’ efficiencies should then be based on either the (expected) most abundant target group or the biggest batch of target taxa showing the same or a similar level of efficiency.

We are aware that the described standardisation of template DNA is not absolutely accurate as there are some steps with small uncertainties. Most of them, however, are also present if a quantified amount of total DNA is used for adjustment. First of all, the precision of this approach is highly dependent on exact pipetting. This problem can be diminished by using pipettes with high precision and accuracy and by precoating the pipette tip before actually taking up the liquid. Pipetting errors will not only impair the measurement of the DNA concentration in the cleaned PCR product with fluorescent dyes, but also affect dilutions and the addition of standardised templates to downstream PCRs. We accounted for potential variation in measured sample volumes during concentration determination by triplicating each sample and averaging the individual measurements. Furthermore, we performed several small dilution steps instead of one or few big ones to keep pipetting errors to a minimum (e.g. 3 × 1:10 is more accurate than 1 × 1:1000). Secondly, a small amount of total DNA, added to the first PCR, is measured alongside with the amplified DNA. However, compared to the quantity of DNA generated during PCR, this amount is negligible, so that amplicon numbers will be only slightly overestimated. This error could be further reduced by separating PCR products on agarose gels and slicing the fragments out prior to cleaning and measurement. Putting these things together, introducing some small errors in template standardisation are inevitable but these should not greatly affect the approach of template standardisation and primer sensitivity adjustment.

### Thermocycling conditions and assay sensitivity

In the current multiplex PCR assay, an annealing temperature of 60 °C proved to be best suited for maximising amplification success as both, at lower and higher temperatures, some fragments were less effectively or not amplified when mixtures of DNA from different taxa were tested. Surprisingly, amplification was successful also at lower/higher temperatures when templates of single taxa were assayed in singleplex as well as in multiplex PCR. The annealing time did affect the sensitivity of the present multiplex PCR system, contradicting the findings of Henegariu *et al.* (1997): by doubling the annealing time from 90 s to 3 min, an increased signal strength was observed. Signal strength derived by 3-min annealing was roughly the same as when 10 times the amount of template was used with 90-s annealing (data not shown).

The sensitivity of the optimised multiplex PCR system proved to be very high, because as little as 20–30 copies of template DNA were sufficient to achieve stable detection. It was possible to detect even lower copy numbers (down to five templates); however, detection reproducibility decreased as expected for samples that contain template DNA close to the detection limit (Sint *et al.* 2011). The sensitivity test again confirmed the balance within the system as the detection limit was very similar for all targets, independent of the co-presence of other target or non-target DNA. This means chances to be amplified in the presented system are equal for all templates during multiplex PCR, avoiding biased detection rates.

Although there are reports that increased cycle numbers could sometimes enhance balanced amplification of different targets (Polz & Cavanaugh 1998; Sipos *et al.* 2007), the mostly negligible effect stands in no relation to the increased probability of producing smears, spurious bands or false-positive results if more than 35 cycles are used (Roux 1995).

## Conclusions

The newly developed method presented here uses quantified PCR products as templates for downstream PCRs and thus allows adjusting the current multiplex PCR system that all fragments are amplified at comparable efficiency. This reduces methodological bias when screening field-derived samples owing to preferred amplification of some fragments and will also give a more realistic snapshot of different targets present in environmental samples. Furthermore, the template copy number for each target is the relevant information needed to compare and balance primer efficiencies not only within but also between studies. The new approach provides a significant improvement of current practices as genome size or variation in copy numbers of multiple-copy genes between individuals, strains or species no longer matters. For new multiplex systems, we recommend to state the minimum number of templates necessary for positive amplification, which will enable comparison of results derived from different studies more easily.

## Supporting Information

**Table S1.** List of species/sequences used to design reverse primer A246.

**Table S2.** List of taxa tested for primer specificity.

As a service to our authors and readers, this journal provides supporting information supplied by the authors. Such materials may be re-organized for online delivery, but are not copy-edited or typeset. Technical support issues arising from supporting information (other than missing files) should be addressed to the authors.
