# Supplementary material for: Advances in multiplex PCR: balancing primer efficiencies and improving detection success
Source: Methods Ecol Evol. 2012 Oct;3(5):898–905. doi: 10.1111/j.2041-210X.2012.00215.x (PMC3573865; doi:10.1111/j.2041-210X.2012.00215.x)
Supplement: Supplementary file 1 [file mee30003-0898-SD1.doc]

Supporting Table 1: List of species/sequences used to design reverse primer A246

| **Species** | **GenBank Accession Number** |
| --- | --- |
| *Callyntrura* sp. | DQ016566 |
| *Crossodonthina koreana* | Z36893 |
| *Crossodonthina tiantongshana* | DQ016557 |
| *Entomobrya dorsosignata* | AY596360 |
| *Folsomia candida* | AY555515 |
| *Folsomia octoculata* | DQ016561 |
| *Friesea japonica* | DQ016558 |
| *Heteromurus nitidus* | AJ605710 |
| *Heteromurus tenuicornis* | DQ016564 |
| *Hypogastrura dolsana* | Z26765 |
| *Hypogastrura duplicispinosa* | DQ016555 |
| *Hypogastrura* sp. | AY596362 |
| *Isotoma viridis* | AY596361 |
| *Isotoma viridis* | AJ605706 |
| *Isotomurus palustris* | DQ016560 |
| *Lepidocyrtus paradoxus* | U61301 |
| *Neanura muscorum* | AY555520 |
| *Neelides minutus* | DQ016567 |
| *Odontella* sp. | DQ016559 |
| *Oncopodura crassicornis* | DQ016563 |
| *Onychiurus hangchowensis* | DQ016554 |
| *Onychiurus yodai* | AY037171 |
| *Papirinus prodigiosus* | DQ016569 |
| *Podura aquatica* | AY596363 |
| *Pseudobourletiella spinata* | DQ016568 |
| *Sinella curviseta* | DQ016565 |
| *Sminthurides aquaticus* | AY596364 |
| *Sminthurinus bimaculatus* | AY555522 |
| *Sminthurus viridis* | AY859604 |
| *Sphaeridia pumilis* | AY145140 |
| *Tomocerus violaceus* | DQ016562 |
| *Tullbergia yosii* | DQ016556 |
