# Supplementary material for: Advances in multiplex PCR: balancing primer efficiencies and improving detection success
Source: Methods Ecol Evol. 2012 Oct;3(5):898–905. doi: 10.1111/j.2041-210X.2012.00215.x (PMC3573865; doi:10.1111/j.2041-210X.2012.00215.x)
Supplement: Supplementary file 2 [file mee30003-0898-SD2.doc]

Supporting Table 2: List of taxa tested for primer specificity. The suffix “mix” represents DNA mixtures, where several individuals were pooled for extraction. Digits in parenthesis denote the number of tested individuals/extracts. Pard – *Pardosa* spp., N.ruf – *Nebria* *rufescens*; O.cas – *Oreonebria* *castanea;* M.gla – *Mitopus* *glacialis*; N.joc – *N*. *jockischii*, N.ger – *N*. *germari*; Col – Collembola; T – target fragment. Digits in the table denote the number of individuals/extracts that produced the respective fragment. For better readability grey shading was added to lines with non-target products.

| **Taxon** | **Pard**  **86bp** | **N.ruf.**  **107bp** | **O.cas**  **129bp** | **M.gla**  **144bp** | **N.joc**  **167bp** | **N.ger**  **198bp** | **Col**  **228bp** |
| --- | --- | --- | --- | --- | --- | --- | --- |
| *Pardosa nigra* | **T** |  |  |  |  |  |  |
| *Pardosa saturatior* | **T** |  |  |  |  |  |  |
| *Pardosa giebeli* | **T** |  |  |  |  |  |  |
| *Nebria rufescens* |  | **T** |  |  |  |  |  |
| *Oreonebria castanea* |  |  | **T** |  |  |  |  |
| *Mitopus glacialis* |  |  |  | **T** |  |  |  |
| *Nebria jockischii* |  |  |  |  | **T** |  |  |
| *Nebria germari* |  |  |  |  |  | **T** |  |
| Sminthuridae (2) |  |  |  |  | **2** |  | **T** |
| Collembola mix (1) |  |  |  |  | **1** |  | **T** |
| Collembola (3) |  |  |  |  | **1** |  | **T** |
|  |  |  |  |  |  |  |  |
| Acalyptratae indet. (1) |  |  |  |  |  |  |  |
| Acari (3) |  |  |  |  |  |  |  |
| Agromyzidae (1) |  |  |  |  |  |  |  |
| Anthomyiidae (5) |  |  |  |  |  |  |  |
| Anthomyiidae mix (3) |  |  |  |  |  |  |  |
| Bibionidae (2) |  |  |  |  |  |  |  |
| Calliphoridae (1) |  |  |  |  |  | **1** |  |
| Cecidomyidae (2) |  |  |  |  | **1** |  |  |
| Chironomidae (5) |  |  |  |  |  |  |  |
| Chloropidae (3) |  |  |  |  |  |  |  |
| Coleoptera (9) |  |  |  |  |  |  |  |
| Dolichopodidae (1) |  |  |  |  |  |  |  |
| Drosophilidae (1) |  |  |  |  |  | **1** |  |
| Empididae (4) |  |  |  |  |  |  |  |
| Enchytraeidae (2) |  |  |  |  |  |  |  |
| Gastropoda (1) |  |  |  |  |  |  |  |
| Homoptera (3) |  |  |  |  |  |  |  |
| Hymenoptera (5) |  |  |  |  |  |  |  |
| Lepidoptera (12) |  |  |  |  |  |  |  |
| *Zygaena* sp. (1) |  |  |  |  |  |  |  |
| Lithobiidae (2) |  |  |  |  |  |  |  |
| Muscidae (8) |  |  |  |  |  |  |  |
| Muscidae mix (4) |  |  |  |  |  |  |  |
| Mycetophilidae (1) |  |  |  |  |  |  |  |
| Phoridae (3) |  |  |  |  |  |  |  |
| Planipennia (3) |  |  |  |  |  |  |  |
| Plecoptera (1) |  |  |  |  |  |  |  |
| Psilidae (1) |  |  |  |  |  |  |  |
| Rhagionidae (1) |  |  |  |  |  |  |  |
| Sciaridae (3) |  |  |  |  |  |  |  |
| Sphäroceridae (1) |  |  |  |  |  |  |  |
| Syrphidae (6) |  |  |  |  | **1** |  |  |
| Tabanidae (1) |  |  |  |  |  |  |  |
| Tachinidae (2) |  |  |  |  |  |  |  |
| Tephridiae (2) |  |  |  |  |  |  |  |
| Tipulidae (4) |  |  |  |  |  |  |  |
| Trichoceridae (2) |  |  |  |  |  |  |  |
| Trichoptera (1) |  |  |  |  |  |  |  |
| unknown insect (1) |  |  |  |  |  |  |  |
| Unknown Larvae (3) |  |  |  |  |  |  |  |
